# Social prescribing in pharmacies: What is it, does it work and what does it mean for Canadian pharmacies?

**DOI:** 10.1177/17151635231216119

**Published:** 2023-12-08

**Authors:** Tarek Hussein, Nia Cartright, Jenny Kirschner, Arun Nadarasa, Adam Pattison Rathbone, Laura Lindsey

**Affiliations:** Weller Pharmacy, Kingston, Ontario; Newcastle University, Faculty of Medical Sciences, School of Pharmacy, Newcastle, United Kingdom; PALS (Pharmacy Addressing Loneliness and Social isolation), Melbourne, Australia; International Social Prescribing Pharmacy Association, Kingsbury, United Kingdom; Newcastle University, Faculty of Medical Sciences, School of Pharmacy, Newcastle, United Kingdom; Newcastle University, Faculty of Medical Sciences, School of Pharmacy, Newcastle, United Kingdom

## What is social prescribing?

In the United Kingdom, the National Health Service (NHS)^1^ defines social prescribing as an initiative enabling the referral of patients to nonclinical support services; it focuses on a holistic approach to care. Globally, social prescribing is defined as “a means for trusted individuals in clinical and community settings to identify that a person has non-medical, health-related social needs and to subsequently connect them to non-clinical supports and services within the community by co-producing a social prescription—a non-medical prescription, to improve health and well-being and to strengthen community connections.”^2^ Social prescribing involves connecting patients to groups, organizations or services that can provide social, emotional or practical support (e.g., to a charity offering advice on debt management for people living with debt or to a lunch club where people living with loneliness can socialize). A key focus of social prescribing is personalized care, which aims to empower individuals to control their physical and mental health using social interventions.^3^ Social prescribing is increasingly seen as complementary to conventional medical and pharmaceutical care and is being integrated into health systems that seek to provide community-based support to personalize care as part of a “business as usual” model.^3^ The relationship between health and social care is intimate, which means social prescribing can be useful for people who experience long-term physical and mental health problems that are rooted in socioeconomic circumstances, such as poor housing, debt and social isolation.^4^ Benefits of linking physical and mental health with social care provides personalized individual opportunities for patients to form social connections, which lead to improved health and well-being outcomes.^5^

Social prescribing referrals are typically made by family physicians, who screen and assess patients who may be eligible for a referral. Suitable patients are referred to a link worker to consult with the patient and collaboratively decide on an appropriate social activity for their needs.^6^ A link worker, sometimes called a community connector or systems navigator, is a person who is usually nonmedically qualified and supports people to work out which community-based support services are available and most suitable for them. Support services such as exercise classes, arts and crafts, volunteering, gardening and cooking lessons, as well as debt management support and accommodation services, provide the tools for patients to make and sustain changes to their lifestyle to support health and well-being.^5^ Such nonclinical support can contribute to the prevention or treatment of chronic diseases and can be as impactful on health outcomes as traditional medication or pharmacological interventions.^2^ Overall, this evidence demonstrates that social prescribing is an emerging health care practice, which presents opportunities for pharmacy teams to support personalized patient care in a new way.

## How effective is social prescribing?

Evidence shows social prescribing can significantly improve health outcomes for individuals.^7-10^ Although evidence evaluating the effectiveness of social prescribing uses short-term evaluations and does not consider other confounding factors that can influence health outcomes,^11^ evidence also suggests social prescribing is effective at reducing demand for conventional health services.^9^ For example, 1 study shows patients engaged in social prescribing visited their family physician 28% less for consultations, and emergency room attendance decreased by 24% compared to patients not engaged with social prescribing. This is important as health systems across the globe struggle to meet patient demand, particularly in areas of high socioeconomic deprivation where health services and resources may be stretched. Pharmacies have been shown to be more densely located in areas of high socioeconomic deprivation compared to other areas,^12,13^ which suggests there may be an opportunity for pharmacies to reduce demand on the health system, as well as improve individual health outcomes for patients, by supporting the implementation of social prescribing.

## Evidence of social prescribing in pharmacy

Social prescribing guidance^1^ identified pharmacies as a suitable setting for social prescribing. Community pharmacies provide much-needed support for patients across a range of age groups, ethnicities, social statuses and physical and mental health conditions.^14^ In 2021, evidence from England reported over a third of patients visited their community pharmacy instead of their family physician practice due to easier access to pharmacies.^14^ The accessibility of community pharmacies is a significant attribute of the professional in any jurisdiction, and with no need to prebook a consultation, pharmacies may be key to increasing access to social prescribing initiatives.^13^ This is important as patient contact in community pharmacies is growing, with a significant push for pharmacists to adopt public health roles in health systems.^15^ Community pharmacy services are increasingly referred to as an “extended scope of practice,” which includes prescribing for common ailments, initiating, renewing and adapting medications and administering injections and immunizations.^16^ This allows for greater contact time with patients, for effective medications counselling and holistic health screening. At the moment, these services are focused on providing conventional physical and mental health care, but they do provide an opportunity to integrate social prescribing in community pharmacies.

Additionally, community pharmacies are private businesses, so funding agendas underpin their services. For example, in some jurisdictions in the United Kingdom, in 2016, community pharmacy funding was cut. This may limit the resources pharmacies have to deliver new initiatives, with evidence in some jurisdictions indicating up to 76% of pharmacies could be forced to reduce services due to poor funding.^17,18^ Meanwhile, other evidence^19^ assessed community pharmacy services’ value to broader health systems. This reported that the value provided through these services was markedly more than received in funding to support them. Funding in community pharmacy is focused primarily on dispensing medications and without significant changes,^19-21^ funding barriers could restrict the role community pharmacy takes in social prescribing. More evidence about the economic impact on pharmacies when offering social prescribing screening and assessment is needed to understand the role pharmacies can have in social prescribing.

## Importance of social prescribing in Canada

Several benefits of social prescribing could be of importance if implemented in Canada. Patients may be able to have their health and care needs met through social prescribing initiatives, reducing demand on already stretched services. One study demonstrated that social prescribing reduced pressures on health care professionals, which could greatly benefit Canada due to the high levels of occupational stress among Canadian physicians.^22^ According to current research, Canadian physicians have experienced stress related to time pressure limitations and procedures imposed on physicians by the government.^22^ Pharmacists adopting social prescribing initiatives in Canada could benefit physicians and improve the working of the health system. Although this might not directly benefit pharmacies, improving the working lives of physicians might enable improved collaboration between physicians and pharmacists—which has been shown to improve pharmacists’ working environments.^23^ Collectively, then, social prescribing is an opportunity to further integrate pharmacies into the health and social care system.

However, implementing social prescribing in Canada could increase pharmacists’ workloads. Pharmacy teams currently experience extreme workloads, which include consultations and patient education.^24^ The addition of social prescribing roles and responsibilities may then be met with some hesitation (or frustration) by the professional teams working in already very busy community pharmacies. Many pharmacy professionals are already informally engaged in social prescribing activities as part of their existing workloads.^10^ This means that the impact of social prescribing on pharmacy teams’ roles and responsibilities may be minimal, as these roles and responsibilities already exist and are well established in pharmacy practice but may need to become more formalized through referral mechanisms to link workers. The formalization of this underrecognized role of pharmacists may then contribute to overall reduced workplace pressures and increased recognition for the profession.

## How to implement social prescribing in Canada

Successful implementation of social prescribing services must be multifactorial, but 2 are critical to initiation—awareness of social prescribing and social prescribing tools. Pharmacy teams must build awareness of social prescribing through educational campaigns and engagement with continuing professional development. Awareness of services and activities patients can be referred to at a local level is also needed.

Once awareness has been achieved, tools to formalize the screening and referral of patients to social prescribing link workers or programs will need to be established. Tools currently exist focusing on specific social determinants of health, for example, on housing^25^ and loneliness,^26^ and include completing forms setting out the health care professional’s concerns or making a phone call to the social prescribing team.^27^ Tools, such as the UCLA Loneliness Scale, are available for health professionals to screen patients to identify who may benefit from referral to a link worker.^26^ The methods or systems implemented in community pharmacies to enable social prescribing must be simple and easy to use, with clear modes of data sharing between community pharmacies and social prescribing link workers and providers. In the United Kingdom, for example, the NHS Long Term Plan aims to recruit additional link workers embedded within regional primary care networks to provide a single point of referral across localities and jurisdictions.^3,28^ However, there is currently limited evidence of pharmacies referring to link workers or social prescribing providers.^10^ New technology and digital health interventions that link to existing technology may have a role here, where clinical decision-making support tools can help screen, identify and refer patients directly to social prescribing providers. For example, social prescribing referrals are being monitored in England using SNOMED codes (linked to existing primary care systems). However, many pharmacy teams do not have the ability to access this system, and therefore the number of referrals by community pharmacy teams is not known or monitored. Further work is required to explore the pharmacy sector’s opportunities to develop social prescribing services quickly and easily. This could include adopting standardized frameworks for screening, assessing and referring patients who may benefit from social prescribing interventions.

## Educational case study exercise

The case study below is provided as an example of a patient case who could present to a community pharmacy and benefit from referral to a social prescribing link worker. After reading it, consider what social prescribing intervention might be useful for this patient within your current practice setting. Then spend approximately 15 to 20 minutes trying to find link workers and social prescribing interventions available in your locality and practice settings. Searches can be completed on Google or other search engines, by reading local, regional or national guidelines and speaking to other health and social care professionals in your area. This activity will help you identify what (if any) social prescribing services are available for you to refer patients in and how you might practise social prescribing screening and referrals in your practice.

## The case

Henry, a 55-year-old patient, enters your pharmacy with a prescription for fluoxetine. He experiences loneliness while working, as he has had to work remotely since the onset of the COVID-19 pandemic and his depression has increased significantly as a result of this. Henry’s drinking has also increased considerably since the pandemic, and he is being treated with disulfiram. After dispensing his fluoxetine, you suggest that Henry speaks with you privately so that you may help further his understanding of the new medication he will be taking to treat his depression.

You give Henry all the details of his new medication, including side effects and instructions on when to take the medication. Instead of sending him on his way after this phase, you also give him additional lifestyle recommendations that could improve his quality of life. Given his current depressive state, drinking habits and loneliness due to the pandemic, you explain how these ailments may impact him in other areas of life. Henry agrees that his feelings of loneliness are a bit debilitating, and you suggest he complete the UCLA Loneliness Scale 4-item version. Upon completing this, it is clear that Henry would be a great candidate for social prescribing.

## Proposed solution

You refer Henry to a social prescribing service in the community, which would assess his needs and co-create a prescription for him by referring him to suitable community resources to benefit Henry’s overall health and decrease loneliness based on what matters to him and what is available in your local area. You provide him with the contact details of your local social prescribing service, which you found on www.socialprescribing.ca/resources, and explain that he is able to contact these organizations directly to participate. You explain that he will work with a link worker or systems navigator. You explain a link worker is someone who knows about the different social programs available and will be able to spend time with him, to help him decide which ones may be best for him. You explain that taking part in the social prescribing interventions is not a guaranteed solution and will not replace his medical care but may make his medical care more effective by improving his depressive state, thus reducing his desire to drink. You offer to meet with Henry again in 4 to 6 weeks to see if he has decided to join any of the groups and what he thinks of them. ■
